# Two New Tetravacant Organometallic Keggin-Type Heteropolyoxomolybdates-Supported Manganese Carbonyl Derivatives

**DOI:** 10.3390/molecules22081351

**Published:** 2017-08-14

**Authors:** Vikram Singh, Yujiao Zhang, Linping Yang, Pengtao Ma, Dongdi Zhang, Chao Zhang, Li Yu, Jingyang Niu, Jingping Wang

**Affiliations:** Henan Key Laboratory of Polyoxometalate Chemistry, Institute of Molecular and Crystal Engineering, College of Chemistry and Chemical Engineering, Henan University, Kaifeng 475004, China; singhvikram001@Gmail.com (V.S.); unscxc@163.com (Y.Z.); linping_1986@126.com (L.Y.); mpt@henu.edu.cn (P.M.); DDZhang@henu.edu.cn (D.Z.); zhangchao@vip.henu.edu.cn (C.Z.); yuli@henu.edu.cn (L.Y.)

**Keywords:** polyoxomolybdates, metal tricarbonyl, electrocatalysts, Keggin structure

## Abstract

Two novel heteropolyoxomolybdate [XMo_8_O_31_]^n−^ (X = Ge(**1**) or P(**2**)) manganese carbonyl derivatives [(CH_3_)_4_N]_6_H_6_{Mn^II^(GeMo_8_O_31_)[Mn^I^(CO)_3_]_2_}_2_·12H_2_O (**1**) and [(CH_3_)_4_N]_4_H_6_{Mn^II^(PMo_8_O_31_)[Mn^I^(CO)_3_]_2_}_2_·14H_2_O (**2**), have been successfully synthesized and characterized in the solid state by single crystal X-ray diffraction, IR and thermogravimetric analysis, and in solution by UV-Vis spectroscopy and electrochemistry. The two polyoxomolybdate-based organometallic compounds **1** and **2** represent rare examples of transition metal sandwich-based polyoxometalate metal carbonyl derivatives (PMCDs), in which the organic-inorganic hybrids are composed of four Mn(CO)_3_^+^ groups symmetrically occupied the tetravacant sites of dimeric heteropolyoxomolybdate {Mn_2_(XMo_8_O_31_)_2_}^n−^ through Mn^I^-O-Mo bonds. The carbonyl functionalized Mn atoms are octahedrally coordinated via three μ_2_-oxygens of the [XMo_8_O_31_]^n−^ unit and three carbonyl carbon atoms. Interestingly, **1** and **2** form a psedocuboidal ring Mn(CO)_3_Mo_3_O_12_ with {Mn(CO)_3_}^+^ occupying the three fold axis of the Mo_3_O_12_ octahedral triad. Beside this, the two centrally placed adjacent Mn^II^ atoms show intramolecular Mn∙∙∙Mn interactions of 3.11 and 3.16 Å in **1** and **2**, respectively. Significant n→π* and O···O intermolecular interactions between the orthogonally aligned adjacent carbonyl groups through the overlap of lone-pair electrons on oxygen atoms with the antibonding orbital (π*) of the adjacent carbony carbon atom of the subsequent units in **1** and **2** were observed. The electrochemical properties of the two compounds were also been investigated.

## 1. Introduction

Polyoxometalates (POMs), are a unique class of metal-oxo clusters, widely studied in different scientific fields such as catalysis, magnetism, medicine and functional materials due to their versatile structures and efficient properties [1,2,3,4,5,6,7,8]. One of the unique attributes of polyoxometalates is their electron acceptor property which allows them to form reduced and mixed valence compounds thus promoting their redox switching behavior [9,10,11]. In recent years, metal carbonyl-functionalized POMs have emerged as an important category of organometallic hybrid clusters that mainly attract attention due to their role in providing new structural classes by altering the electronic and structural features of the basic transition metal-substituted POMs structural motifs [12,13]. Therefore, immobilization of metal carbonyl units onto the polyoxometalate surface plays a significant role in obtaining functional compounds which can enjoy the dual advantages [14]. Metal carbonyl-substituted POMs have long been studied due to their high catalytic activity, particularly in many organic reactions [15,16,17]. Since the advent of the first POM-incorporating carbonyl cobalt derivative reported by Knoth in 1979 [18], POM-based metal carbonyl derivatives have emerged as compounds of significant importance and thus are being increasingly studied.

In recent years, a number of POM-based metal carbonyl derivatives (PMCDs) particularly ones based on classical Keggin- and Dawson-type structures have been consistently of interest [19,20,21,22] (Table 1). In this direction, our group has also contributed substantially and first explored the synthesis and electrochemical properties of octatungstate- and octamolybdate-supported tricarbonyl metal derivatives: [(H_2_X_8_O_30_){M(CO)_3_}_2_]^8−^ (M = Mn^I^ and Re^I^: X = W, Mo) in 2011 and 2013, respectively [23,24]. Recently, we also reported heteropolytungstate- and isopentatungstate-supported metal carbonyl derivatives and their electrocatalytic application for nitrite reduction [25,26]. Previously, the structural analogues of these metal carbonyl compounds based on heteropolyoxotungstates and heteropolyoxomolybdates were also reported [27,28,29]. However, transition metal sandwiched heteropolyoxomolybdates incorporating metal cartbonyl units are still very few. In this context, and our interest in exploring and developing organometallic-based POM clusters, encouraged us to successfully obtain two new tetravacant Keggin-type heteropolyoxomolybdate metal carbonyl derivatives: [(CH_3_)_4_N]_6_H_6_{Mn^II^(GeMo_8_O_31_)[Mn^I^(CO)_3_]_2_}_2_·12H_2_O (**1**) and [(CH_3_)_4_N]_4_H_6_{Mn^II^(PMo_8_O_31_)[Mn^I^(CO)_3_]_2_}_2_·14H_2_O (**2**), whose structures revealed four organometallic {Mn(CO)_3_}^+^ units symmetrically placed onto the three-fold axis of the Mo_3_O_12_ octahedral triad, with two additional octahedrally-coordinated Mn^II^ atoms that lay within the sandwich-type heteropolyoxomolybdates.

The structures **1** and **2** represent rare examples of hybrid POMs having both organometallic and transition metal-induced structural and electronic features. The detailed studies related to their synthesis, and interesting redox behaviour due to active Mn^II^/Mn^I^ redox couple, were undertaken and the results of these investigations are now described here.

## 2. Results and Discussion

### 2.1. Synthesis

**Scheme 1:**
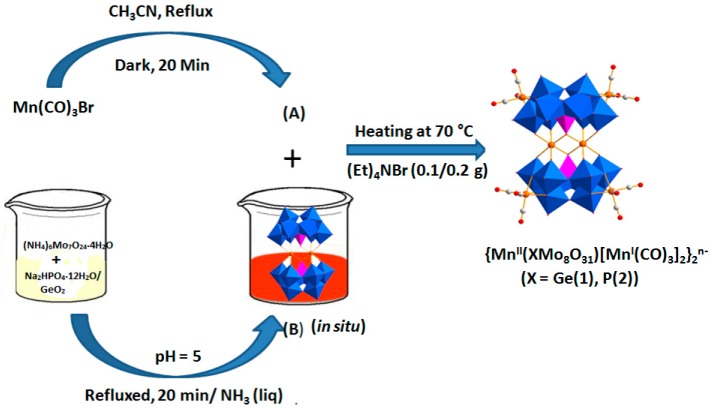
Preparation of novel PMCDs **1** and **2** via a two-step reaction protocol. Colour code: O, red balls; C, grey balls; Mn/Mn, orange balls; Mo_6_ octahedra, blue; XO_4_ (X = P/Ge) tetrahedral, purple.

The synthesis of heteropolyoxomolybdate-supported tricarbonyl metal derivatives **1** and **2** was achieved using (NH_4_)_6_Mo_7_O_24_·4H_2_O as the starting material via a two-step reaction protocol in a mixed solution of water/acetonitrile and water/methanol for **1** and **2**, respectively (Scheme 1). A series of parallel experiments showed that the reaction pH values play an important role in the formation of these two new organometallic compounds. The syntheses of **1** and **2** were conducted in a weakly acidic aqueous solution (pH 5–6), and crystals of suitable size were obtained at pH 5 by adding aqueous ammonia solution. Moreover, slight deviations from this pH value hamper the successful crystallization of **1** and **2**. Based on previous reports, it could be envisaged that the organometallic metal substituents, particularly the metal carbonyl cations, attach less well onto the anionic oxygen atoms of the POM framework due to the steric hindrance of the organic group [14,27], thus it is quite difficult to obtain high nuclear PMCDs.

In line with the above facts, we successfully obtained POM-supported Mn(CO)_3_^+^ with additionally sandwiched Mn^II^ atoms in between the two [XMo_8_O_31_]^n−^ subunits that form the [Mn_4_(CO)_6_Mn_2_(XMo_8_)_2_]^n−^ (X = Ge(**1**), P(**2**)) structural framework. It is noted that the crystals of **1** and **2** are sensitive to weathering and thus were converted into powders on exposure to air.

### 2.2. Crystal Structures

**Figure 1:**
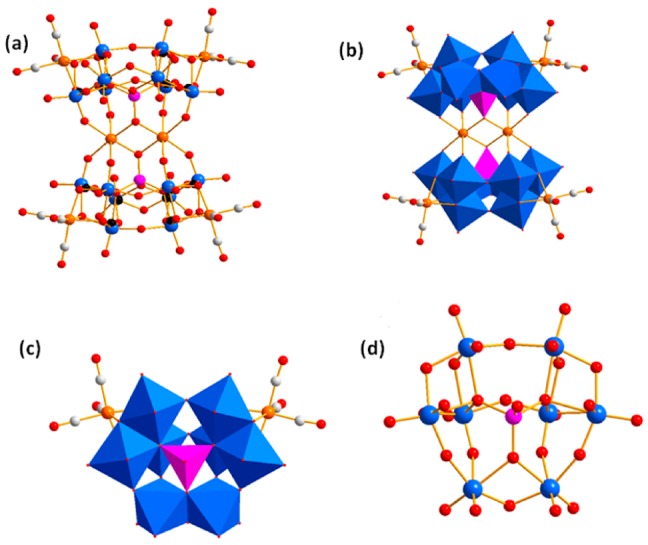
(**a**) Ball-and-stick representation of **1** and **2**; (**b**) Polyhedral representation of **1** and **2**; (**c**) {Mn(CO)_3_}^+^ grafted anions **1** and **2**; (**d**) Ball-and-stick representation of anions [XMo_8_O_31_]^n−^ (X = Ge(**1**) or P(**2**)). Colour code: O, red; C, grey; Mo, blue; Ge/P, pink; Mn, orange.

Crystallographic data, structure refinement, and selected bond lengths and angles for **1** and **2** are presented in Tables S1–S3 of the Supporting Information. Single-crystal X-ray diffraction analyses revealed that **1** and **2** are almost isomorphous. Compounds **1** and **2** both crystallize in the triclinic space group *P-1* with similar structural features, composed of two symmetrical [(XMo_8_O_31_){Mn^I^(CO)_3_}_2_]_2_^n−^ (X = Ge(**1**) or P(**2**)) units joined by a central pair of Mn^II^ ions, resulting in a sandwich structural core (Figure 1a). Interestingly, each central manganese atom bonded symmetrically with six bridging oxygen atoms; amongst them four µ_2_-O atoms bridged Mo^VI^ to the Mn^II^ atoms within each [XMo_8_O_31_]^n−^ unit. Beside this, the two centrally placed adjacent Mn^II^ are linked via two µ_3_-O atoms thus connected to each other at Mn∙∙∙Mn distances of 3.11 and 3.16 Å with ∠Mn(3)-O(31)-Mn(3): 92.77° and 94.02° in **1** and **2**, respectively. The atoms Mn(3)/O(9), O(12), O(31) and O(31) lay in one plane with O(9)-Mn(3)-O(12) and O(31)-Mn(3)-O(31) of 90.77 and 87.23°, respectively, along with the two axially placed µ_2_O(11)/O(12) atoms that complete the octahedral coordination geometry around the Mn^II^ atoms. In addition, there are two carbonyl-substituted Mn^I^ atoms that are octahedrally coordinated through three μ_2_-O atoms of the [XMo_8_O_31_]^n−^ unit and three carbon atoms from three carbonyl groups (Mn-O: 2.020(7)–2.063(6) Å, Mn-C: 1.747(14)–1.8156(11) Å). Here, the three carbon atoms take up one triangular facet of the octahedron together with the three oxygen atoms in opposite positions thus the {Mn(CO)_3_}^+^ occupies the three fold axis of the Mo_3_O_12_ octahedral triad to form the pseudocuboid Mn(CO)_3_Mo_3_O_12_. The two symmetrically-placed cuboids get connected via two µ_2_-O atoms to form dual-core manganese metal centers in both **1** and **2** (Figure 1b). Notably, each [(XMo_8_O_31_){Mn^I^(CO)_3_}_2_]^n−^ (X = Ge(**1**) or P(**2**)) units can be regarded as a tetravacant Keggin-[XMo_8_O_31_]^n−^ fragment carrying two {Mn(CO)_3_}^+^ groups (Figure 1c), while the [XMo_8_O_31_]^n−^ fragment is derived from the well-known saturated Keggin polyanion by removal of a edge-shared Mo_3_O_13_ triad and a MoO_6_ octahedron from the 60° vertically placed Mo_3_O_13_ triad.

**Figure 2:**
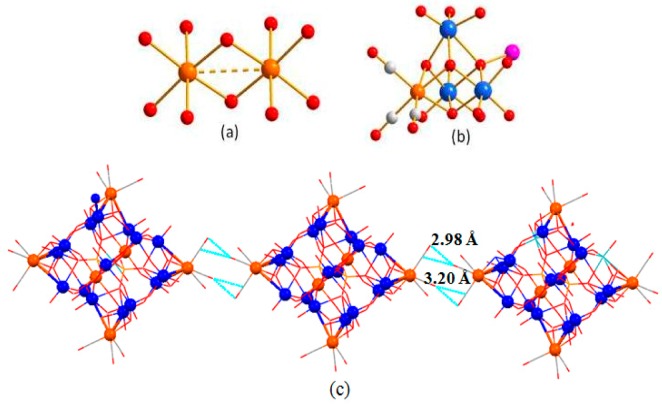
(**a**) Mn∙∙∙Mn interactions of 3.11 and 3.16 Å in **1** and **2**; (**b**) Pseudocuboids Mn(CO)_3_Mo_3_O_12_ formation in **1** and **2**; (**c**) Supramolecular framework through non-covalent n→π* and O···O interactions in **1** and **2**. Colour code: O, red balls; C, grey balls; Mn, orange balls; Mo, blue; XO_4_ (X = P/Ge) tetrahedral, purple.

In **1** and **2**, the oxygen atoms are classified into five groups according to their different coordination environments, as in anion **1**: (1) 16 terminal oxygen atoms emanating from one Mo and six carbonyl carbon atoms [Mo = O: 1.688(8)–1.744(6) Å]; (2) 11 μ_2_-O atoms bridged between the two Mo atoms [Mo-(μ_2_-O):1.849(7)–2.209(5) Å; (3) one μ_3_-O bonded to two Mo and a germanium atom, [Mo-(μ_3_-O): 2.201(6)–2.210(6) Å; (4) two μ_4_-O atoms bridging three Mo atoms and a germanium atom, [Mo-(μ_4_-O): 2.273(6)–2.372(5) Å; Ge-O: 1.704(5)–1.781(5) Å. Interestingly, clusters **1** and **2** sustain a supramolecular framework through non-covalent n→π* and O···O interactions. Significant n→π* intermolecular interactions are observed between the orthogonally aligned adjacent carbonyl groups through the overlap of lone pair electrons on oxygen atoms with the antibonding orbital (π*) of the adjacent carbony group of the subsequent units. The distances between the donor and acceptor atoms in **1** and **2** are 3.20 Å, in agreement to the previous report [30]. Beside this, the carbonyl oxygen atoms also sustain non-covalent chalcogen-chalcogen O···O intermolecular interactions with distances of 2.98 Å (Figure 2) [31,32]. It can be observed that the intermolecular O···O distances is shorter then the sum of the van der Waals radii (ΣR_VdW_) of oxygen atoms. A close look into the structures of **1** and **2** reveals fascinating intramolecular Mn∙∙∙Mn interactions of 3.11 Å, in **1**, which are relatively stronger as compared to 3.16 Å in **2**. It is significant to note that **1** and **2** are amongst the few carbonyl metal derivatives within the Keggin type polyoxometalates carrying four {Mn(CO)_3_}^+^ moieties grafted onto the anionic POM surface. It is noteworthy that {[XMo_8_O_31_]^n−^ (X = Ge(**1**) or P(**2**)) (Figure 1d) is although similar to the tetravacant Keggin polyanion [SiW_8_O_31_]^10−^ [19] and [PW_8_O_31_]^9−^ [29] units, but still relatively less explored. Importantly, the current contribution of [Mn_4_(CO)_6_Mn_2_(XMo_8_)_2_]^n−^ (X = Ge(**1**), P(**2**)) are the new addition that may enrich the subclass of POM-supported carbonyl clusters based on [XMo_8_O_31_]^n−^ (X = Ge(**1**) or P(**2**)) polyanions.

The BVS values for the sandwich Mn atoms in **1** and **2** are presented in Table 2. It is obvious that the four Mn atoms connected to carbonyl groups are in +1 oxidation states.

### 2.3. Electrochemistry

**Figure 3:**
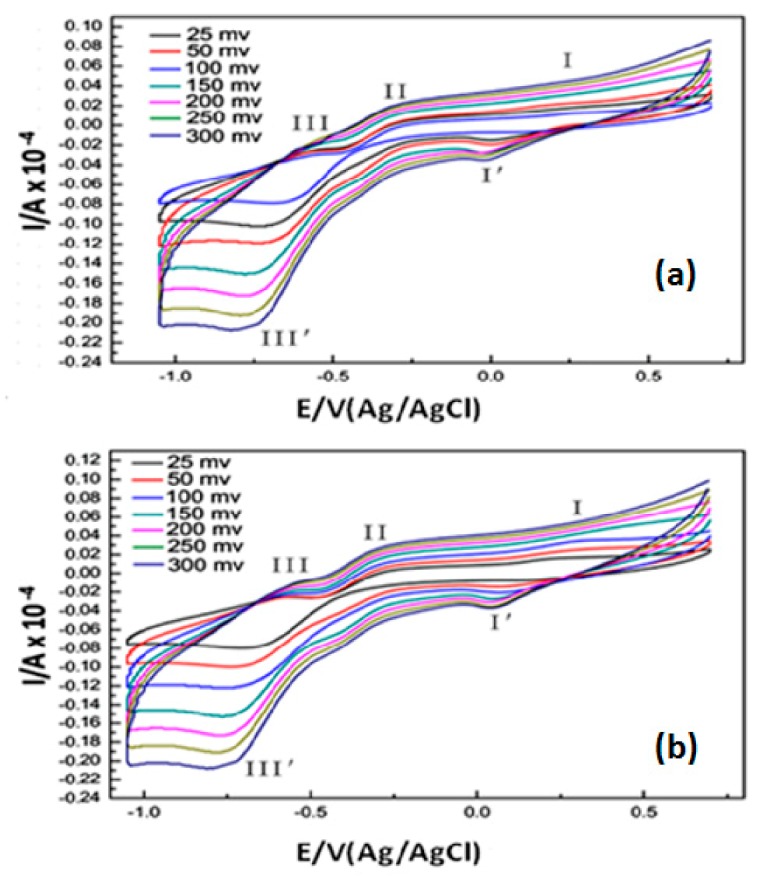
The CV curves of **1** (**a**) and **2** (**b**) in the mixed solvent of CH_3_CN–Na_2_SO_4_ (1:3, volume ratio) (0.4 mol·L^−1^) at different scan rates (from inner to outer: 25, 50, 100, 150, 200, 250, 300 mV·s^−1^).

**Figure 4:**
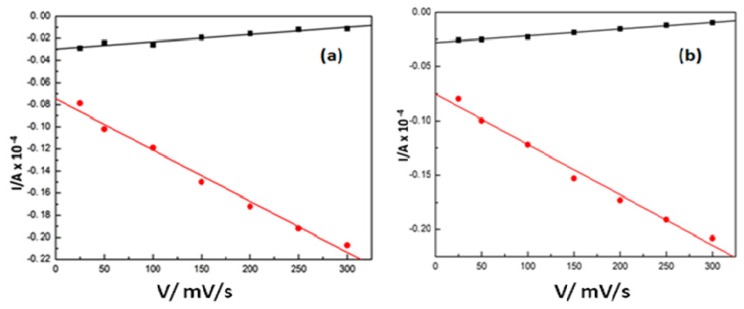
The variation of the third couple peak currents (red: anodic, black: cathodic) against the square root of the scan rates from 50 to 300 mV·s^−1^ of **1** (**a**) and **2** (**b**). The working electrode is glassy carbon and the reference electrode is SCE.

The results of CV experiments of **1** and **2** are quite similar (Figure 3a,b). At the potential range of **1** (Figure 3a), one irreversible oxidation peak and two pairs of quasi-reversible redox waves appeared with the E_1/2_(V) at −0.636 (III–III′) and 0.322 (I–I′) that correspond to the redox processes based on Mo^VI^ atoms and {Mn(CO)_3_}^+^ pendant groups, respectively. The redox wave (I–I′), although not clearly discerned in the case of **1**, could be seen for **2** and is mainly attributed to the oxidation/reduction of Mn^I^/Mn^II^, while the Mo^VI^-based waves are located in the negative potential region. The influence of the scan rate on the current of **1** and **2** has also been studied under the same reaction conditions and the results illustrate the variation of cathodic peak currents of the Mo^VI^-based waves with different scan rates. When the scan rate is varied from 25 to 300 mV s^−1^, the peak currents are proportional to the root of the scan rates in both cases, which suggests that a surface-controlled electron-transfer process is occurring at **1** and **2**, respectively (Figure 4).

The detection and removal of nitrite ions from the environment and foodstuffs is a matter of great concern. Considerable efforts have been made to introduce various electrocatalysts that could be beneficial in enhancing the elecrocatalytic reduction process of nitrites. Previously, electrocatalytic nitrite reduction by various transition metal-substituted heteropolyoxotungstate-based electrocatalysts were performed in CH_3_COONa + CH_3_COOH buffer solution [33,34,35,36]. A few reports on electrocatalytic reduction using Na_2_SO_4_ + CH_3_CN solution were reported using heteropolytungstate as electrocatalyst, but PMCD-based electrocatalysts in mixed solvent, i.e., CH_3_CN + Na_2_SO_4_ are still not explored.

**Figure 5:**
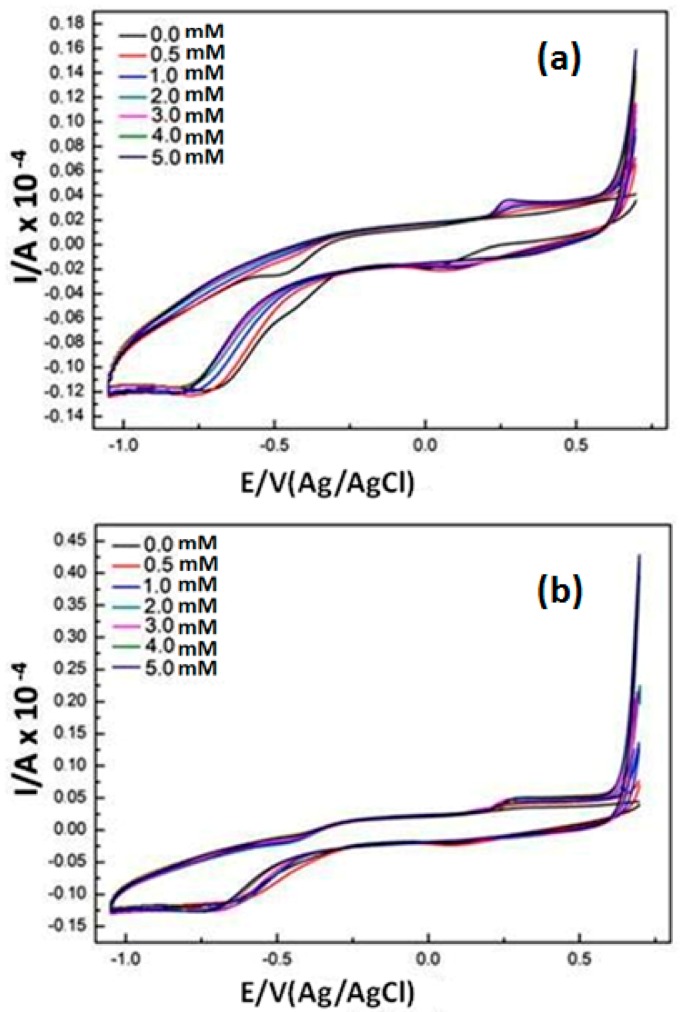
The CVs of **1** (**a**) and **2** (**b**) in the mixed solvent of CH_3_CN–Na_2_SO_4_ (1:3, volume ratio) at the concentration of 1 × 10^−5^ mol·L^−1^, scan rate of 100 mV·s^−1^ with gradual addition of NaNO_2_.

**Figure 6:**
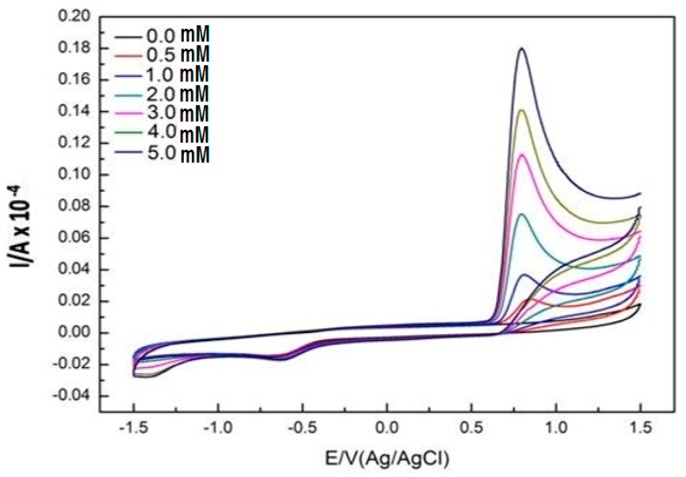
The CVs of 0.0–5.0 mM NaNO_2_ solution in the absence of **1** and **2** recorded at 100 mV·s^−1^ scan rate. The working electrode is glassy carbon and the reference electrode is SCE.

The electrocatalytic nitrite reduction for **1** and **2** were investigated under the same reaction conditions as those employed in the CV studies. Like previous reports on electrocatalytic nitrite reduction, the gradual additions of different concentration of NaNO_2_ to 1 × 10^−5^ mol L^−1^ of **1** and **2** were carried out and the changes in the redox potentials of **1** and **2** were observed (Figure 5). The cyclic voltammograms depicts the variation of cathodic peak currents of the Mo^VI^-based wave. On addition of NO_2_^−^, the reduction peak of Mo centers show a certain shifting in a negative potential direction, while the corresponding oxidation peak disappears gradually, indicating that compounds **1** and **2** play a significant role in the electrocatalytic reduction of nitrite (Figure 4). Moreover, an irreversible oxidation peak appears at the positive potential region with the gradual addition of nitrite. This peak corresponds for NO_2_^−^ as confirmed from the cyclic voltammogram spectra obtained in the absence of compounds **1** and **2** (Figure 6).

### 2.4. UV–Vis Spectroscopy

**Figure 7:**
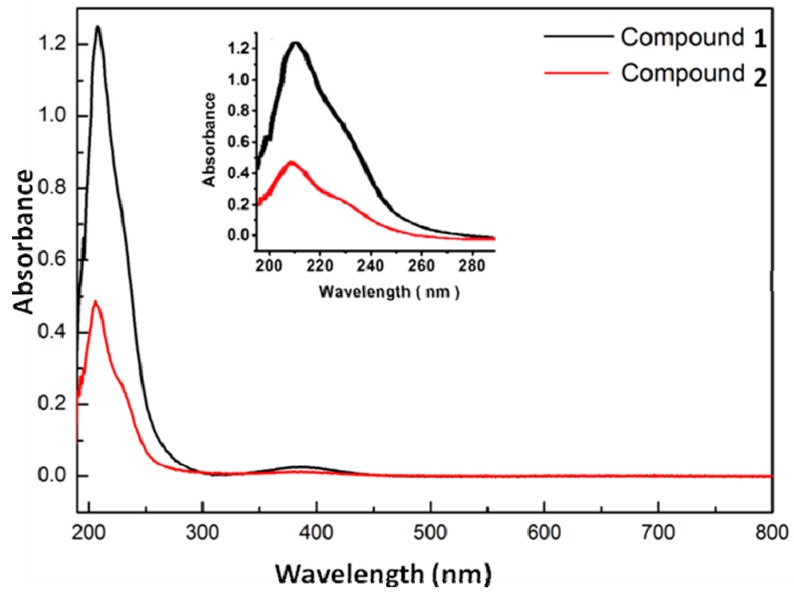
The UV-Vis spectra of **1** and **2** in the mixed solvent of CH_3_CN/H_2_O (1:3, volume ratio).

**Figure 8:**
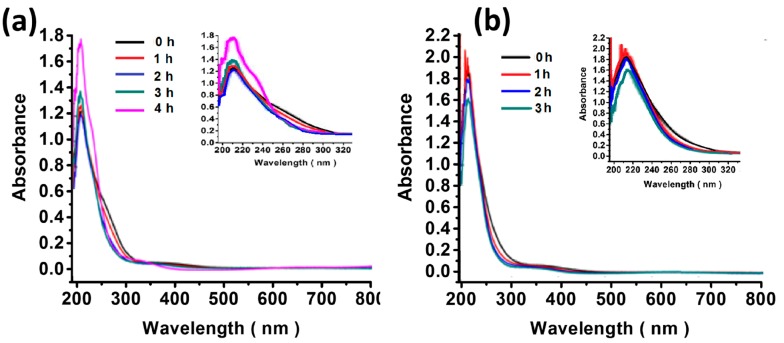
The aging of the solutions of **1** (**a**) and **2** (**b**) detected by in situ obtained UV-Vis spectra changes.

The UV–Vis spectra were monitored to investigate the properties of **1** and **2** in the mixed solvent CH_3_CN–H_2_O (1:3, volume ratio) in the range of 200–500 nm. The clusters show similar characteristic UV–Vis curves, thus supporting the isostructural nature and similar bonding patterns in **1** and **2** (Figure 7). The band for **1** and **2** that appears at 206 nm can be attributed due to the O_t_→Mo charge transfer transitions [37], while, the broad absorption band at ca. 388 nm can be assigned to Mn (π)→CO (π*) transitions, thus illustrating the presence of manganese carbonyl groups (Figure 7) [38,39,40,41]. In order to investigate the stability of **1** and **2** in solution, the UV–Vis spectra were recorded in regular intervals for up to 4 h in a mixed solvent mixture (Figure 8) and it is corroborated that compound **1** remains stable for about 3 h at room temperature in a dark environment, while compound **2** lost its stability after 2 h.

### 2.5. IR Spectroscopy

**Figure 9:**
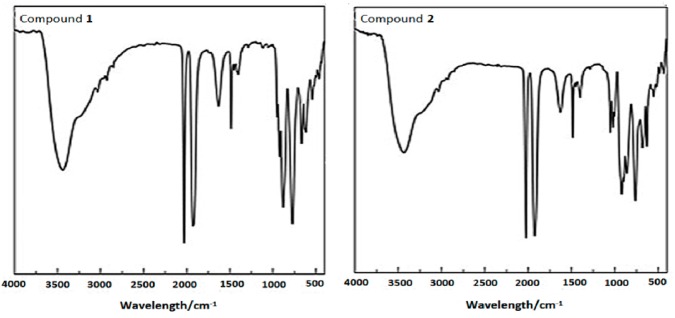
FT-IR spectra (KBr pellets) of **1** (**a**) and **2** (**b**).

The IR spectra of **1** and **2** (Figure 9) show similar characteristic stretching vibrations in the region of 400–4000 cm^−1^. Furthermore, strong bands for **1** and **2** appear at 936, 905, 773, 667, 615 cm^−1^ and 940, 905, 769, 684, 628, 595, 545 cm^−1^ that could be assigned for the ν (Mo–O), ν (X–O) (X = Ge or P) for **1** and **2**, respectively. The asymmetric and symmetric stretch vibrations of C=O groups appear as strong bands at ca. (2025, 1925) and (2035, 1900) cm^−1^ for **1** and **2**, respectively. Similar bands are observed for a number of C_3V_ carbonyl metal complexes in the carbonyl stretching region [42]. The resonance at about 3437 cm^−1^ is attributed to –OH and –NH stretching and the flexural vibration waves of –OH and –NH at 1621 and 1485 cm^−1^ are observed meanwhile. In addition, bands at 1048 and 1020 cm^−1^ in compound **2** assigned to the ν (P–O) vibrations.

### 2.6. Thermogravimetric Analysis

**Figure 10:**
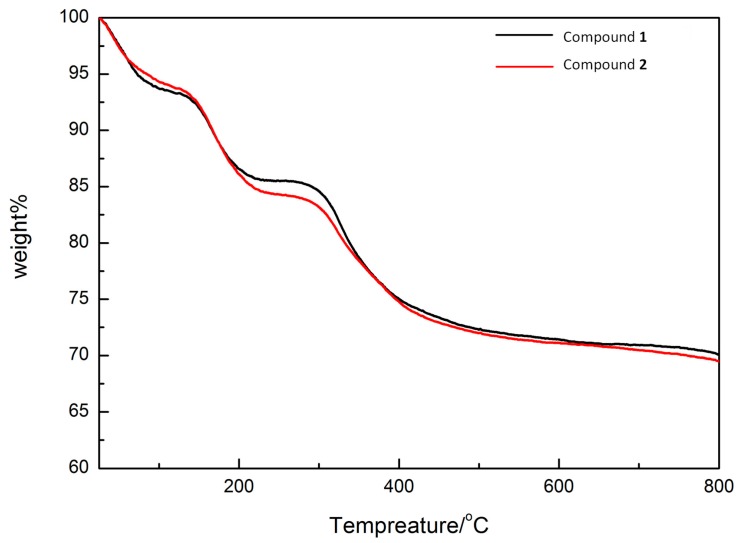
Thermogram of **1** and **2**.

The thermogravimetric (TG) analyses of **1** and **2** have been performed under a nitrogen atmosphere in the 25–800 °C temperature range at a slow heating rate of 10 °C/min. The TG curves show three step mass losses in the above temperature range (Figure 10), giving a total loss of 33.70% (calcd. 32.77%) for **1** and 35.68% (calcd. 33.50%) for **2**.

For **1**, the first stage spectral change from 25 to 110 °C is attributed to the loss of twelve lattice water molecules, and the observed weight loss of 5.80% is comparable with the calculated value (5.40%). The second stage weight loss of 8.50% occurs between 110 and 270 °C, which could be due to the loss of twelve carbonyl groups (calcd. 8.40%). The third stage with the weight loss of 14.10% from 270 to 800 °C can be attributed to the removal of six tetramethylammonium cations and six protons (in the form of constitutive water molecules) (calcd. 13.78%). In the case of **2**, the TG curve follows a similar decomposition trend as in **1**, the first stage from 25 to 120 °C is ascribed to the loss of fourteen lattice water molecules, and the observed weight loss of 6.28% is consistent with the calculated value of 6.61%. The second stage 9.47% weight loss that appears occurs between 120 and 240 °C, and may be due to the removal of twelve carbonyl groups (calcd. 8.82%). The third stage that appears with a weight loss of 11.95% from 240 to 800 °C shows the removal of four tetramethyl-ammonium cations and six protons (in the form of crystallized water molecules) (calcd. 10.70%).

## 3. Experimental Section

### 3.1. Materials and General Methods

All the chemical reagents were commercially available and used without further purification. IR spectra were obtained from solid sample pelletized with KBr on a 170 SXFT-IR spectrometer (Nicolet, Madison, WI, USA) in the range 400–4000 cm^−1^. UV-Vis. spectra were recorded on a U-4100 spectrometer (Hitachi, Tokyo, Japan). Elemental analyses (C, H and N) were conducted on a 2400-II CHNS/O analyzer (Perkin-Elmer, 940 Winter Street Waltham, MA, USA). Inductively coupled plasma (ICP) spectra were obtained on a Perkin-Elmer Optima 2000 ICP-OES spectrometer. TGA experiments were performed under a N_2_ atmosphere on a TGA/SDTA851 instrument (Mettler-Toledo, Sonnenbergstrasse 74, Greifensee, Switzerland ) with the heating rate of 10 °C min^−1^ from 25 to 800 °C. All electrochemical measurements were performed at room temperature in a standard three-electrode cell connected to a LK98 microcomputer-based electrochemical system (LANLIKE, Tianjin, China). A freshly cleaned glassy carbon disk electrode (3 mm diameter) was used as a working electrode, a platinum wire served as the counter electrode and an Ag/AgCl as the reference electrode. For electrochemical experiments 10^−5^ M solutions of **1** and **2** in CH_3_CN–Na_2_SO_4_ (0.4 mol·L^−1^) (1:3, volume ratio) were prepared and the electrochemical experiments were performed in the dark.

### 3.2. Synthesis of [(CH_3_)_4_N]_6_H_6_{Mn^II^(GeMo_8_O_31_)[Mn^I^(CO)_3_]_2_}_2_·12H_2_O *(**1**)*

Mn(CO)_5_Br (0.137 g, 0.498 mmol) was refluxed in CH_3_CN (7.0 mL) in the dark for 20 min under a N_2_ atmosphere and then the reaction mixture was cooled to room temperature (Solution A). In a separate reaction, (NH_4_)_6_Mo_7_O_24_·4H_2_O (0.453 g, 0.5 mmol), GeO_2_ (0.023 g, 2.5 mmol) and Mn(Ac)_2_·4H_2_O (0.613 g, 2.5 mmol) were added to distilled water (15 mL) with subsequent addition of ammonia (0.2 mL) and refluxed for 25 min with constant stirring (Solution B). Next reaction mixture A was added slowly to B at 70 °C for 1 h, followed by the addition of tetramethylammonium bromide (0.1 g). The reaction mixture thus obtained was further stirred for 20–25 min at 70 °C, and then cooled to room temperature. The reaction mixture was filtered, the filtrate was allowed to stand in the dark for slow evaporation. The dark red rod shaped crystals of **1** were isolated after some days. (Yield: ca. 8% based on Mn(CO)_5_Br). Elemental analysis (%) calcd. for **1**: C, 10.80; H, 2.57; N, 2.10; Ge, 3.63; Mo, 38.32; Mn, 8.24. Found: C, 10.83; H, 2.09; N, 2.16; Ge, 3.54; Mo, 38.55; Mn, 8.46; IR (KBr, cm^−1^): 3437 (vs), 2025 (s), 1925 (vs), 1621 (vs), 1485 (s), 1402 (s), 1248 (m), 1201 (w), 1008 (w), 936 (s), 905 (s), 769 (m), 684 (s), 595 (s), 541 (s), 493 (s) (Figure S1, ESI).

### 3.3. Synthesis of [(CH_3_)_4_N]_4_H_6_{Mn^II^(PMo_8_O_31_) [Mn^I^(CO)_3_]_2_}_2_·14H_2_O *(**2**)*

Mn(CO)_5_Br (0.137 g, 0.498 mmol) was refluxed in CH_3_CN (7.0 mL) in the dark for 20 min under a N_2_ atmosphere and then cooled to room temperature (Solution A). In another separate reaction, (NH_4_)_6_Mo_7_O_24_·4H_2_O (0.618 g, 0.5 mmol), Na_2_HPO_4_·12H_2_O (0.618 g, 2.5 mmol), and Mn(Ac)_2_·4H_2_O (0.613 g, 2.5 mmol) were dissolved in distilled water (15 mL) and the reaction mixture was refluxed for 25–30 min at 70 °C then cooled to room temperature and filtered (Solution B). To this filtrate (Solution B), slow addition of Soluion A was performed under constant stirring at 70 °C for 30 min, then the reaction mixture was allowed to attain room temperature followed by the addition of tetramethylammonium bromide solution (0.5 mmol) and subsequent stirring for another 1–2 min at room temperature. The reaction mixture was finally filtered and was allowed to stand in the dark for slow evaporation. The red block shaped crystals of **2** were isolated after some days. Yield: ca. 28% based on Mn(CO)_5_Br. Elemental analysis (%) calcd. for **2**: C, 8.83; H, 2.17; N, 1.47; P, 1.64; Mn, 8.73; Mo, 40.63. Found: C, 8.94; H, 2.12; N, 1.58; P, 1.57; Mn, 8.63; Mo, 40.52. IR (KBr, cm^−1^): 3437 (vs), 2035 (s), 1900 (vs), 1620 (vs), 1480 (s), 1422 (s), 1240 (m), 1205 (w), 1048 (w), 1020(w), 940 (s), 905 (s), 760 (m), 689 (s), 598 (s), 545 (s), 490 (s) (Figure S1, ESI).

### 3.4. Crystallography

Crystallographic data for **1**, **2** were all collected at 296 K using an Bruker Apex II diffractometer equipped with a CCD bidimensional detector with the graphite monochromated Mo Kα radiation (λ = 0.71073 Å) with an optical fiber as the collimator. The absorption correction was based on multiple and symmetry-equivalent reflections in the data set using the SADABS program (Sheldrick, G. M. SADABS-Bruker AXS area detector scaling and absorption, version 2008/2001; University of Göttingen: Göttingen, Germany, 2008). The structures were solved by direct methods and refined using full-matrix least squares on F^2^. All calculations were performed using the SHELXTL-97 program package [43]. No hydrogen atoms associated with water molecules were located from the difference Fourier map. Hydrogen atoms attached to carbon and nitrogen atoms were geometrically placed. All hydrogen atoms were refined isotropically as a riding mode using the default SHELXTL parameters. All non-hydrogen atoms were refined anisotropically except for some water molecules.

## 4. Conclusions

In this contribution we have reported the syntheses and structure elucidation of two new heteropolyoxomolybdate-supported tetracarbonyl metal derivatives **1** and **2** by conventional methods. The study demonstrates the feasibility of the formation of some new POM-based metal carbonyl derivatives (PMCDs) through simple inorganic salts. The two polyoxomolybdate-supported organometallic compounds **1** and **2** contain tetracarbonyl functionalized {Mn(CO)_3_}^+^ groups with octahedrally coordinated manganese atoms via three μ_2_-oxygen of the [XMo_8_O_31_]^n−^ (X = Ge(**1**), P(**2**)) units and three carbon atoms from carbonyl groups. Interestingly, **1** and **2** form psedocuboidal Mn(CO)_3_Mo_3_O_12_ rings where {Mn(CO)_3_^+^} occupies the three-fold axis of the Mo_3_O_12_ octahedral triad. Beside this, the two clusters show intramolecular Mn∙∙∙Mn interactions of 3.11 and 3.16 Å for **1** and **2**, respectively. Significant n→π* and O···O intermolecular interactions between the orthogonally aligned adjacent carbonyl groups through the overlap of lone-pair electrons on oxygen atoms with the antibonding orbital (π*) of the adjacent carbonyl carbon atom of the subsequent units in **1** and **2** were observed. Electrochemical experiments have shown that compounds **1** and **2** exhibit efficient electrocatalytic activity for successful nitrite reduction. The interesting structures presented in this contribution may be helpful for synthetic chemists to obtain heterometallic PMCDs by using different transition metal salts and organometallic precursors. This work presented here widens the scope of the synthesis of novel organometallic-based PMCDs that may possibly act as electrocatalysts in various reactions.

## Figures and Tables

**Table 1:** Summary of recently reported PMCDs.

Year	Formula	Reference
2008	Cs_6_[SiW_11_O_39_Ru^II^(CO)]·8H_2_O	[19]
2011	(NH_4_)_4_[H_4_{[H_2_Mo_8_O_30_][Mn(CO)_3_]_2_}]·12H_2_O	[24]
	(NH_4_)_4_[{H_6_Mo_8_O_30_}^−^{Re(CO)_3_}_2_]·14H_2_O	
2013	H_6_[Na(H_2_O)_5_]_2_{[H_2_W_8_O_30_][Mn(CO)_3_]_2_}·13H_2_O	[23]
	H_2_[Na(H_2_O)_5_]_2_[Na(H_2_O)_4_]_2_[Na(H_2_O)_2_]_2_{[H_2_W_8_O_30_][Re(CO)_3_]_2_}	
	[Na(H_2_O)_5_]_2_[Na_2_(µ_2_-H_2_O)_2_(H_2_O)_4_]_2_[Mn(H_2_O)_2_]{[H_2_W_8_O_30_][Mn(CO)_3_]_2_}	
2016	KH[(CH_3_)_4_N]_3_{[Re(CO)_3_]_4_[(μ_2_-OH)(μ_3_-O)(W_5_O_18_)]}·6H_2_O	[26]
2013	Na_11_H[Sb_2_W_20_O_70_{Re(CO)_3_}_2_]·34H_2_O	[21]
	Na_11_H[Bi_2_W_20_O_70_{Re(CO)_3_}_2_]·33H_2_O	
	K_9_Na_3_[Sb_2_W_20_O_70_{Mn(CO)_3_}_2_]·32H_2_O	
	K_9_Na_3_[Bi_2_W_20_O_70_{Mn(CO)_3_}_2_]·32H_2_O	
2013	[P_2_W_17_O_61_{Re(CO)_3_}_3_{ORb(H_2_O)}(μ_3_-OH)]^9−^	[14]
2014	[P_4_W_35_O_124_{Re(CO)_3_}_2_]^16−^	[22]
2012	K_8_[(OC)_3_Mn(A-α-H_2_GeW_9_O_34_)]·10H_2_O	[20]
	K_8_[(OC)_3_Mn(A-α-H_2_SiW_9_O_34_)]·11H_2_O	
2014	[(CH_3_)_4_N]_5_H_23_{(PW_11_O_39_){Re(CO)_3_}_3_(µ_3_-O)(µ_2_-OH)]_4_·24H_2_O	[27]
2015	{[PMo_3_O_16_][Re(CO)_3_]_4_}^5−^	[25]
2016	[(M4(H_2_O)_10_(XW_9_O_33_)_2_{Mn(CO)_3_}_2_]^n−^ (X = Sb/Bi; Mn = Mn/Mn_3.5_W_0.5_)	[28]
2015	(NH_4_)_3_H_3_[{Mn(CO)_3_}(Mn(H_2_O)_2_(Mn(H_2_O)_3_)(TeW_9_O_33_)]_2_·31H_2_O	[29]

**Table 2:** The bond valence of atoms in **1** and **2**.

Atoms	1	2
Mo	5.99–6.09	6.17–6.62
Mn	2.30	2.42

## Supplementary Materials

Supplementary materials are available online. Tables S1–S3 (Bond lengths and bond angles, Single crystal X-ray data). CCDC 1054346(**1**)–1054347(**2**) contains the supplementary crystallographic data for this paper. These data can be obtained free of charge via http://www.ccdc.cam.ac.uk/conts/retrieving.html (or from the CCDC, 12 Union Road, Cambridge CB21EZ, UK; Fax: +44 1223336033; Email: deposit@ccdc.cam.ac.uk).
