# Supplementary material for: Two New Tetravacant Organometallic Keggin-Type Heteropolyoxomolybdates-Supported Manganese Carbonyl Derivatives
Source: Molecules. 2017 Aug 14;22(8):1351. doi: 10.3390/molecules22081351 (PMC6152162; doi:10.3390/molecules22081351)
Supplement: Supplementary file 1 [file molecules-22-01351-s001.pdf]

Supporting Information

Vikram Singh, Yujiao Zhang, Linping Yang, Pengtao Ma, Dongdi Zhang, Chao Zhang, Li Yu, Jingping Wang* and Jingyang Niu*

Henan Key Laboratory of Polyoxometalate Chemistry, Institute of Molecular and Crystal Engineering, College of Chemistry and Chemical Engineering, Henan University, Kaifeng, Henan 475004 (P.R. China)

E-mail: jyniu@henu.edu.cn

Two new tetravacant organometallic Keggin-type heteropolyoxomolybdates-supported manganese carbonyl derivatives

Section S1. General characterization

1. Single crystal X-Ray and selected bond lengths and bond angles details (Table S1-S3).

1. Single crystal X-Ray diffraction:

Crystallographic data for **1–2** were all collected at 296 K using a Bruker Apex II diffractometer equipped with a CCD bidimensional detector with the graphite monochromated Mo Kα radiation (λ = 0.71073 Å). The absorption correction was based on multiple and symmetry-equivalent reflections in the data set using the SADABS program. The structures were solved by direct methods and refined using full-matrix least squares on F^2. All calculations were performed using the SHELXTL-97 program package. No hydrogen atoms associated with water molecules were located from the difference Fourier map. Hydrogen atoms attached to carbon and

nitrogen atoms were geometrically placed. All hydrogen atoms were refined isotropically as a riding mode using the default SHELXTL parameters. All non-hydrogen atoms were refined anisotropically except for some water molecules. Crystallographic data, structure refinements and selected bond lengths and bond angles for **1** and **2** are summarized in Table S1-S3.

Table S1. Crystallographic Data for **1** and **2**

	**1**	**2**
Empirical formula	$C_{36}H_{102}Mn_6Mo_{16}N_6O_{86}Ge_2$	$C_{28}H_{82}Mn_6Mo_{16}N_4O_{88}P_2$
formula weight	4005.08	3809.55
space group	P-1	P-1
crystal system	Triclinic	Triclinic
a (Å)	14.0027(10)	13.766(7)
b (Å)	14.2208(10)	13.860(7)
c (Å)	18.2196(14)	17.728(9)
α (deg)	109.3510(10)	108.699(8)
β (deg)	106.3180(10)	106.446(9)
γ (deg)	98.7090(10)	98.798(10)
V(Å^3)	3163.2(4)	2960(3)
Z	1	1
crystal size (mm^3)	0.55 x 0.27 x 0.17	0.15 x 0.09 x 0.07
D_{calcd} (g cm^{-3})	2.101	2.134
μ [mm^{-1}]	2.664	2.373
GOF on F^2	1.127	1.011
R_1, [I >2σ(I)]	0.0577	0.0839
wR_2	0.1843	0.1857
R_1(all data)	0.0753	0.2104
wR_2	0.2027	0.2188

Table S2. Bond lengths and bond angles for **1.**

**Bond Lengths (Å)**			
Mn(1)-O(17)	2.035	Mn(1)-C(1)	1.796
Mn(1)-O(12)	2.006	Mn(1)-C(2)	1.807
Mn(1)-O(20)	2.061	Mn(1)-C(3)	1.769
Mn(2)-O(22)	2.010	Mn(2)-C(4)	1.875
Mn(2)-O(23)	2.046	Mn(2)-C(5)	1.746
Mn(2)-O(24)	1.975	Mn(2)-C(6)	1.780
Mn(3)-O(11)	2.156	**Bond angles (◦)**	
Mn(3)-O(12)	2.062	O(9)-Mn(3)-O(12)	90.77
Mn(3)-O(9)	2.087	O(31)-Mn(3)-O(31)	87.23

Mn(3)-O(10)	2.155	O(31)-Mn(31)-O(9)	91.75
Mn(3)-O(31)	2.173	O(31)-Mn(31)-O(12)	90.28
P(1)-O(28)	1.529	Mn(3)-O(31)-Mn(31)	92.77
P(1)-O(29)	1.547		
P(1)-O(30)	1.507		
P(1)-O(31)	1.517		

Table S3. Bond lengths and bond angles for **2**.

**Bond lengths(Å)**			
Mn(1)-O(17)	2.063	Mn(1)-C(1)	1.810
Mn(1)-O(12)	2.028	Mn(1)-C(2)	1.797
Mn(1)-O(20)	2.043	Mn(1)-C(3)	1.796
Mn(2)-O(22)	2.039	Mn(2)-C(4)	1.816
Mn(2)-O(23)	2.057	Mn(2)-C(5)	1.747
Mn(2)-O(24)	2.020	Mn(2)-C(6)	1.762
Mn(3)-O(11)	2.231	**Bond Angles (◦)**	
Mn(3)-O(12)	2.154	O(12)-Mn(3)-O(31)	90.96
Mn(3)-O(9)	2.149	O(31)-Mn(3)-O(31)	87.23
Mn(3)-O(10)	2.223	O(31)-Mn(3)-O(31)	85.98
Mn(3)-O(31)	2.130	O(11)-Mn(3)-O(12)	84.73
Ge(1)-O(28)	1.775	Mn(3)-O(3)-Mn(31)	94.02
Ge(1)-O(29)	1.781		
Ge(1)-O(30)	1.754		
Ge(1)-O(31)	1.704		
Mn(2)-O(22)	2.039		
Mn(2)-O(23)	2.057		
